# Systems-level chromosomal parameters represent a suprachromosomal basis for the non-random chromosomal arrangement in human interphase nuclei

**DOI:** 10.1038/srep36819

**Published:** 2016-11-15

**Authors:** Sarosh N. Fatakia, Ishita S. Mehta, Basuthkar J. Rao

**Affiliations:** 1 Department of Biological Sciences, Tata Institute of Fundamental Research, Mumbai, Maharashtra 400005, India; 2 UM-DAE Centre for Excellence in Basic Sciences, Biological Sciences, Kalina campus, Santacruz (E), Mumbai, Maharashtra 400098, India

## Abstract

Forty-six chromosome territories (CTs) are positioned uniquely in human interphase nuclei, wherein each of their positions can range from the centre of the nucleus to its periphery. A non-empirical basis for their non-random arrangement remains unreported. Here, we derive a suprachromosomal basis of that overall arrangement (which we refer to as a CT constellation), and report a hierarchical nature of the same. Using matrix algebra, we unify intrinsic chromosomal parameters (e.g., chromosomal length, gene density, the number of genes per chromosome), to derive an extrinsic effective gene density matrix, the hierarchy of which is dominated largely by extrinsic mathematical coupling of HSA19, followed by HSA17 (human chromosome 19 and 17, both preferentially interior CTs) with all CTs. We corroborate predicted constellations and effective gene density hierarchy with published reports from fluorescent *in situ* hybridization based microscopy and Hi-C techniques, and delineate analogous hierarchy in disparate vertebrates. Our theory accurately predicts CTs localised to the nuclear interior, which interestingly share conserved synteny with HSA19 and/or HSA17. Finally, the effective gene density hierarchy dictates how permutations among CT position represents the plasticity within its constellations, based on which we suggest that a differential mix of coding with noncoding genome modulates the same.

During the interphase stage of the cell cycle in human nuclei, a chromosome is confined to a restricted physical location that is referred to as a chromosome territory (CT)^1^, which occupies a small fraction of the nuclear volume. Qualitative and quantitative microscopy studies have reported their anisotropic positioning, whose overall format is referred to as the radial arrangement (reviewed in refs 2, 3, 4). During interphase, CTs not only occupy non-random locations in the nucleus, but also intermingle via dynamical looping interactions at their borders^5^, while their relative macroscopic position largely persists with respect to other CTs (reviewed in refs 2, 3, 4), however their individual positions are dynamic during cell cycle and other biological contexts such as DNA damage response^6^ and starvation response^7^. In any arrangement of forty-six CTs, which we refer to as a constellation, territories may couple to their neighbours by loci-mediated contacts^5^,^8^ and also via lamin and other nuclear-matrix mediated interactions^9^,^10^,^11^. Fluorescent *in situ* hybridization (FISH) based light microscopy mapping of a clonal population of normal human cells during interphase have reported preferential locations of solo CTs^12^,^13^,^14^, and as a conglomerate of two or more CTs^6^,^15^,^16^. However, within the human nucleus, significant diversity in preferential positions have also been reported for chromosomes 5, 14 and 21 (abbreviated as chr5, chr14 and chr21) even in identical cell types^6^,^12^,^15^,^16^. Moreover, it is also observed that the preferred positions of CTs such as chr1, chr5, chr11, chr21 and chrY, are markedly different in diverse cell types^12^,^15^,^16^. High-throughput sequencing with chromosome conformation capture (Hi-C) experiments^17^ and Hi-C-based tethered chromosome conformation capture (TCC) experiments^18^ have reported the contact probability distributions of cross linked chromatin, based on which a map of inter-CT neighbourhoods is computed to represent the ensemble average of most likely CT constellations. Most importantly, a single-cell Hi-C study^19^ has reported variability in the physical structure and spatial conformation of individual CTs, again using a clonal population, suggesting that the discovery of rare CT constellations may be most challenging from a limited ensemble size. Therefore, in the context of plasticity within a constellation, a physical basis for non-random radial arrangement is essential.

Chromosome territories are spatially and temporally regulated and it has been reported that normal chromosomal function significantly impacts their position and vice versa (reviewed in refs 20, 21, 22, 23, 24). For example, in an asynchronous population of human dermal fibroblasts, it has been shown that four distinct CTs undergo statistically significant displacement in the nucleus (relocate from their original position to a new one) after inducing DNA double strand breaks by cisplatin (DNA damaging agent) treatment^6^. In a significant population of those nuclei, chr17 and chr19 relocated from the interior of the nucleus to the periphery, while chr12 and chr15 were displaced oppositely, from the periphery towards the interior^6^. The average Chromosomal territories (CT) arrangement observed in an ensemble of DNA-damaged cells after DNA-damage repair were found to be similar to the control ensemble of undamaged cells, demonstrating that reversible changes are associated with DNA damage response^6^. Independent studies, in cancers and genetic instability disorders, have reported that DNA double-strand breaks lead to translocations specifically among closely located CTs^25^. Therefore, it is concluded that spatially non-random CT constellations directly influence their functions and translocation propensities^26^. As CT constellations significantly differ in tumor versus normal cells, by virtue of their inter-CT distances^25^,^26^,^27^,^28^, it is critical to investigate the fundamental basis of the same. An *in vitro* study has demonstrated that the spatial position of a human chromosome is conserved even in human-mouse hybrid nuclei, sustaining an overall radial form of chromosomal arrangement^29^. Interestingly, yet another independent study has demonstrated that while nearly fifty percent of human chromosomes demonstrated a conserved spatial arrangement in such human-mouse hybrid nuclei, other chromosomes follow the localization pattern specified by their syntenic murine chromosomes^30^. All these observations seem to point towards an organization that subsumes several CTs non-randomly into another level beyond that of solitary CTs such that a non-random clustering of CTs defines neighbourhood of a given CT constellation. We refer to such a scale as “suprachromosomal” in the context of spatial arrangement of CTs in the nucleus. Therefore, we surmised that if there exists a species-independent suprachromosomal basis for the non-random anisotropic CT arrangement, such an organization must be influenced by macroscopic chromosomal parameters and remain tenable across disparate vertebrates.

To understand a physical basis of the radial chromosomal arrangement of forty-six CTs in the human nucleus, traditional microscopy-based methods are supplemented with *in silico* approaches involving polymer-based models (reviewed in refs 31, 32, 33, 34). It is established that polymers can faithfully represent chromosomal topology and reproduce clustered spatial arrangement^35^,^36^. Based on the evidence that polymers without excluded volume and negligible intermingling generate a radial form of arrangement, it has been demonstrated that topological constraints govern territorial clustering^37^,^38^. Moreover, it has been shown that even entropic (nonspecific) factors can lead to anisotropic constellations that may self-organize into spatial patterns, as observed in the ‘beads on string’ model^39^ and the ‘strings and binders switch’ model^40^. More recently, the role of polymer loops and entropic factors have been used to model and describe CT arrangements in prokaryotes and eukaryotes^41^,^42^. In order to derive a theoretical basis of the radial CT arrangement, these *in silico* methods have used both impermeable as well as permeable polymers (phantom)^38^. Based on early investigations^43^,^44^,^45^, using intrinsic parameters of the human coding genome and by incorporating coding, noncoding and pseudogenes, a recent study has also successfully modeled radial CT arrangement in human nuclei (with “gene rich” CTs such as chr19 at the nuclear centre)^46^. More recently, it has been reported that topologically associating domains (TADs)^47^, the minimal physical entities of CTs, fold with invariant boundaries across different cell types^48^, and in turn provide a physical basis of hierarchical folding encompassing ‘domains-within-domains’ or metaTADs^49^. However, a non-empirical basis of the physical organization representing the plasticity and hierarchy of CT constellations, at a suprachromosomal-level, has not been reported yet. Consequently, the *in silico* methods have not addressed the plethora of CT constellations that exist *in vitro*.

In the context of an interactive chromosomal milieu within the human nucleus, here we report a non-empirical “suprachromosomal” basis for CT constellations describing their plasticity. We derive systems-level mathematical and physical constraints that are exclusively genome-based, and describe a theory for self-organized CT constellations. Using this theoretical basis, we confirm the plasticity of interphase CT positioning in the nucleus as obtained from FISH^6^,^12^,^13^,^14^,^15^,^16^ and Hi-C derived inter-CT maps^17^,^18^ in human interphase nuclei. Importantly, as our method represents a conglomerate of “suprachromosomal” entities, we do not incorporate the polymer-based constraints or topology to model the radial arrangement of forty-six CTs within the human nucleus. From an evolutionary perspective, we also corroborate the veracity of our predictions, using independently reported FISH results from disparate vertebrates: chimpanzee^50^, mouse^51^, pig^52^ and chicken^53^. Based on these disparate genomes, we report a theoretical basis for the hierarchy and plasticity within each of their CT constellation, predict the inner CTs in their respective nuclei and subsequently corroborate their identity using previously published results.

## Results

The traditional schematic of the human genome is the linear DNA sequence of nucleotides, which includes the twenty-four different chromosomes in sequential order chr1, chr2, …, chr21, chr22, chrX, and chrY. In this report, we consolidate the intrinsic parameters of all chromosomes in an abstract 24D vector space and mathematically derive extrinsic suprachromosomal parameters (representing a suprachromosomal biological crosstalk or mathematical coupling among different CTs). This abstract formalism is used to identify systemic extrinsic constraints among the forty-six CTs in an interactive milieu, which constitute a non-random CT constellation. Moreover, we also show that the suprachromosomal coupling supports an identical hierarchical arrangement within every unique constellation, which arises along with permissible degeneracy (ambiguity due to CT permutations), as in a classical statistical ensemble of accessible CT constellations.

### Systems-level suprachromosomal coupling coefficient represents effective gene density

Here, we report a mathematical formalism to depict all unique suprachromosomal entities in an *in vivo* context, which is distinct from the traditional *in vitro* context, where individual CTs are accessed without the influence of other CTs (Materials and Method). For *in vitro* representation we symbolically denote the different chromosomes of the human genome (chr1, chr2… chr21, chr22, chrX and chrY) as *C*_*j*_ (where 1 ≤ *j* ≤ *N* = 24). We derived all extrinsic suprachromosomal parameters that exhaustively characterized all possible *in vivo* CT constellations. We hypothesized that for each nearest-neighbour pair 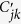 (*C*_*j*_ and *C*_*k*_ CT pair) the composite effective gene count (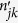) or paired chromosome’s gene count (PCGC) was mathematically coupled with an effective length 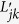 via the relative effective gene density (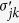) as:


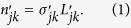


First, we defined a dimensionless PCGC parameter 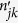 as the harmonic mean of the total number of annotated genes in the coding and noncoding genome for 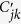. The harmonic mean (as opposed to a geometric mean or an arithmetic mean) is an ideal statistic that represents two or more very diverse number of genes per chromosome, as it gives lower weightage to very high values. Therefore, the effective gene count for a given 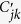 was represented (in terms of its intrinsic parameters *n*_*j*_ and *n*_*k*_) as:


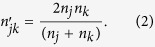


Next, to suitably characterize the length of 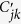, we defined its effective length 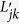 as the harmonic mean of its intrinsic lengths (*L*_*j*_ and *L*_*k*_):


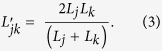


Furthermore, representing 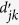 as a harmonic mean of intrinsic average gene densities:


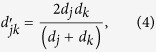


and using equations (1, 2, 3, 4), we derived the extrinsic suprachromosomal coupling coefficient as:


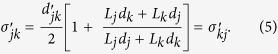


This suprachromosomal spatial coupling coefficient parameter 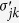 has the physical dimensions of gene density and is therefore referred to as its effective gene density. Hypothetically, if *C*_*j*_ and *C*_*k*_ are physically nearest-neighbours, then 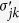 is the mathematical coupling among CTs, as if it is a suprachomosomal unit 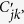 an *in vivo* entity with an effective length 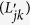 and effective number of genes 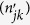. To assess t contributions of all suprachromosomal entities in a genome, we normalized 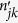 in equation (1) using two genome-specific normalization constants: 
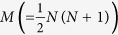
, 
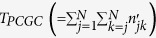
, and expressed equation (1) as a percentage:


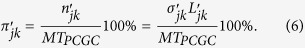


Here, using the intrinsic chromosomal parameters for the diploid human genome, we have formulated a method to set up all possible paired combinations (*M* = 300) from the 24 (=*N*) distinct CTs. Using these macroscopic chromosomal parameters, we had no means to distinguish maternal versus paternal chromosomes.

### The sum total of all the extrinsic effective gene density parameters in the human genome greatly exceed intrinsic ones

Our minimalistic *N*-dimensional (24D) vector model invokes suprachromosomal entities 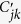 instead of solitary CTs. In equation (5), the derived effective gene density for 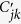 had a conventional term (*L*_*j*_*d*_*j*_) and an unconventional “mixed” term (*L*_*j*_*d*_*k*_), presenting a systems-level coupling of CTs. All effective gene density (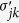) parameters were represented using the effective gene density matrix – a *N* × *N* real symmetric matrix 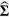 (Materials and Methods), with 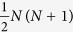 unique elements. Each element of the matrix (which is an extrinsic or intrinsic parameter) was derived using macroscopic intrinsic chromosomal parameters. Of the derived 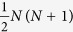 unique parameters, *N* were previously known intrinsic ones (diagonal elements) and 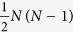 were the extrinsic ones (off-diagonal elements). Hence, in the human nucleus, where the haploid number *N* = 24, the maximum number of all possible extrinsic systems-level parameters is twelve times greater than that found in a more familiar *in vitro* model. The theory presented here exploits mathematical couplings among homologous CTs (modeled by a conventional term) and non-homologous pairs (modeled by a “mixed” term). Therefore, using first principles, we have derived a large number of mathematical constraints, at the pan-nuclear level, among putative pairs of homologous and non-homologous chromosome partners.

### The effective gene density matrix constrains the human CT arrangement

In this formalism, we have represented extrinsic suprachromosomal coupling coefficients, 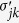, as off-diagonal (*j, k*) elements of the systems-level coupling matrix 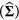 (Materials and Methods). Using equations (4) and (5), each matrix element 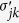, for 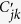, was derived as:


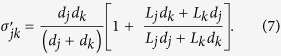


**Figure 1:**
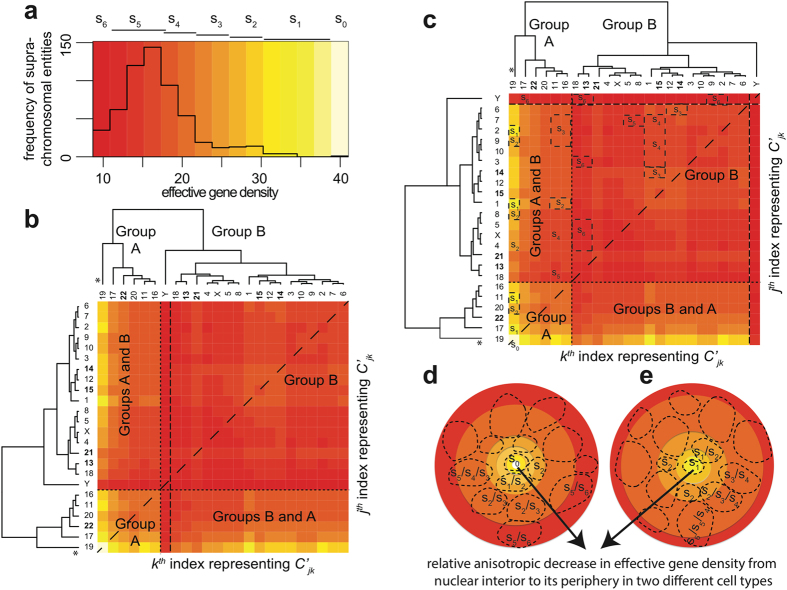
The effective gene density obtained from extrinsic suprachromosomal entities of the human genome. A histogram of effective gene density from all unique chromosome pairs 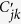 is represented in (**a**). The histogram colour key is encoded at the top of panel (a) as s_6_/s_5_ (dark/light red), s_4_/s_3_ (dark/light orange), s_2_/s_1_ (dark/light yellow) and s_0_ (off-white) as effective gene density values increase along the x-axis. A hierarchically clustered heatmap of the effective gene density matrix from all 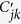 chromosome pairs indexed by labels *j* and *k* on the y- and x-axes respectively is shown in (**b**). The values of effective gene density from 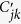 and 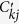 are identical, indicated by a dashed diagonal line, and the horizontal and vertical dashed lines segregate suprachromosomal pairs when both *C*_*j*_ and *C*_*k*_ belong to Group A, or Group B, or their admixture. Here, chromosome Y (denoted chrY, from Group B) is highlighted by two vertical dashed lines, which represent its unique position in the overall hierarchy that is dominated by chromosome 19 (marked “*”). The colour key for effective gene density values in (**a**, **b**) are consistent. The five acrocentric CTs: chromosomes 13–15, 21 and 22 are represented with bold labels. Exploiting the degeneracy of chrY, with respect to all other chromosomes of the human genome, a mathematically equivalent heatmap is generated in panel (**c**). Suprachromosomal entities coloured s_0_ to s_6_ gradient in (**a**) are mapped as boxes in (**c**). For example, s_1_ is represented by chr19 paired with chr2/chr1/chr11/chr17, s_2_ represented by chr19 when paired with chr9/chr8/chr4/chr20 and so on. Similarly, deeper hues from orange to red represent a progressively larger pools of degenerate suprachromosomal pairs with similar effective gene density. Hypothetically, cohorts of suprachromosomal entities s_0_–s_6_ may be used to recreate a 3D spherical representation of CT arrangement for cell type-specific human nuclei in a 2D rendition (panels **d, e**), arranged with relatively high effective gene density contributors such as s_0_/s_1_/s_2_ toward the interior of the nuclei and the relatively low effective gene density contributors such as s_6_/s_5_ toward the periphery as indicated by the arrow.

The large diversity in the effective gene density matrix is represented by a histogram (Fig. 1a) and hierarchically clustered heatmap (Fig. 1b). The histogram for effective gene density is asymmetric, skewed toward higher values. The highest effective gene density for 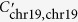 is statistically significant from the histogram mean (its difference from the mean value is 4.7 times the root-mean-square deviation of the histogram). Effective gene density substantiates that for a given CT there may be variability in its extrinsic gene density that is contingent on the neighbourhood of CTs (left-most column and lower-most row in Fig. 1b). For example, effective gene density coupling for 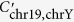 is much lower (less than fifty percent) than the coupling for 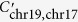.

Exploiting the hierarchical position of chrY, with respect to all other chromosomes of the human genome, a mathematically equivalent dendrogram is generated in Fig. 1c. There, all chrY pairs were removed from their original format in Fig. 1b, and juxtaposed to the extreme right and on the top. Such an equivalent rendition is one of numerous possible dendrograms, which can maintain the original suprachromosomal hierarchy in effective gene density (as vertical branch lengths are preserved), although CT neighbourhoods may swap with each other. Horizontal branches connote a Boolean type of mother/daughter hierarchical relation among branch leaves (which represent CTs) in a given clade (group or subgroup as the case may be). Therefore changing their absolute lengths does not alter the overall hierarchy. The coupling between CTs assessed via relative effective gene density is implied by the vertical height of the dendrograms. Apart from the off-white colour (denoted by s_0_) of the heatmap grid for chr19 homologs, we have coarsely delineated six colour categories (s_1_– s_6_) to describe effective gene density of suprachromosomal entities. Multiple suprachromosomal entities are represented on the heatmap (boxes s_0_–s_6_ in Fig. 1c), which capture the degeneracy of effective gene density in the light of CT positions in the interphase nucleus. We illustrate that a plethora of permutations exist, leading to plasticity in CT constellations, for cell type-specific instances, as represented by cartoons in Fig. 1d,e. Progressively different colours, such as dark orange to red to dark red, reflect progressively larger pool of degenerate CT pairs (as seen from the peak of histogram in Fig. 1a and the relatively high number of 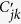 grids with orange-red colour). This implies that if extrinsic (effective) gene density constraints were to dictate the preferential positioning of CTs, then co-occurrence of chr17 and chr19 as a pair would be strongly biased in contrast to that of the chr19 and chrY pair, thereby implying preferred physical clustering between chr17 and chr19 as opposed to chr19 and chrY, leading to the precursor stage of suprachromosomal organization defined earlier.

**Figure 2:**
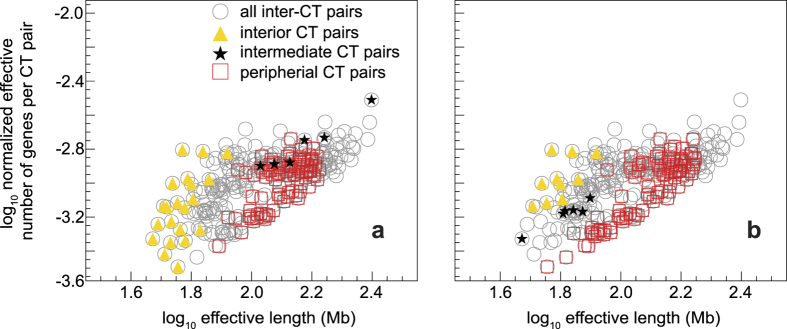
Normalized effective number of genes versus effective length for suprachromosomal pairs. The graph of normalized effective number of genes (*π*′_*jk*_) for every 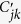 pair versus their effective length (*L*′_*jk*_) for all 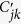 is shown as a circle (open circle). Panel (**a**) represents the preferential CT location data from fibroblast nuclei and (**b**) represents lymphocyte data. The coordinates representing 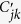 pairs, wherein both CTs are from nuclear interior (triangle), periphery (box), and spatially intermediate (star) regions, are superimposed over those representing all pairs (open circle). The ones shown in the open circle that do not overlap with either a triangle, box, or star, represent those pairs where CTs belong to a different mix of spatial categories. Only unambiguous spatial CT positions from Table S1 are represented.

Using the genome-specific normalized PCGC values (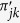), we wanted to determine if neighbourhood CT effects could be used to delineate spatial CT arrangements. The *in vitro* spatial positions of individual CTs were already known from a high volume microscopy study performed using dermal fibroblast nuclei^6^. Using the most probable CT position from microscopy-based data for fibroblast nuclei (ref. 6 and summarized in Table S1), we computed 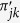 versus 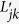 (effective length) for cases in which *C*_*j*_ and *C*_*k*_ were both exclusive to the nuclear interior, the periphery, and spatially intermediate to the interior and the periphery. Such a scatter-plot of 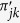 versus 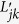 (Fig. 2a), whose composition is fibroblast cell type-specific, confirms the segregation among paired CTs that are in the inner core versus the periphery of the nucleus, suggesting a hierarchy in paired chromosome gene count (PCGG) versus effective length for 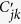. The scatter-plot retrieved for the fibroblast also describes an intermediate category, which depends on whether *C*_*j*_ and *C*_*k*_ are in the interior or peripheral part of the nucleus. However, it is important to note that the PCGC data cluster of interior versus periphery is different in lymphocytes (ref. 12 and summarized in Table S1), leading to commensurate changes in the intermediate category (Fig. 2b). Interestingly, data from those 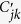 that do not overlap with instances where both CTs are confined to the interior/intermediate/periphery is also represented in the scatter-plot. A CT from any one of the above three spatial zones coupled with another CT from a different zone constitutes the non-overlapping category (Fig. 2). Therefore, we hypothesize that a systematic hierarchy and degeneracy in spatial CT arrangement is obtained when inter-chromosomal coupling via effective gene density is computed. We surmise, while the scatter-plot in Fig. 2 is representative of the overall allowable pattern, the cell type-specificity dictates the details of CT positioning between the nuclear interior versus intermediate versus peripheral zones.

### Hierarchy of effective gene density from the human genome supports CT constellations

**Figure 3:**
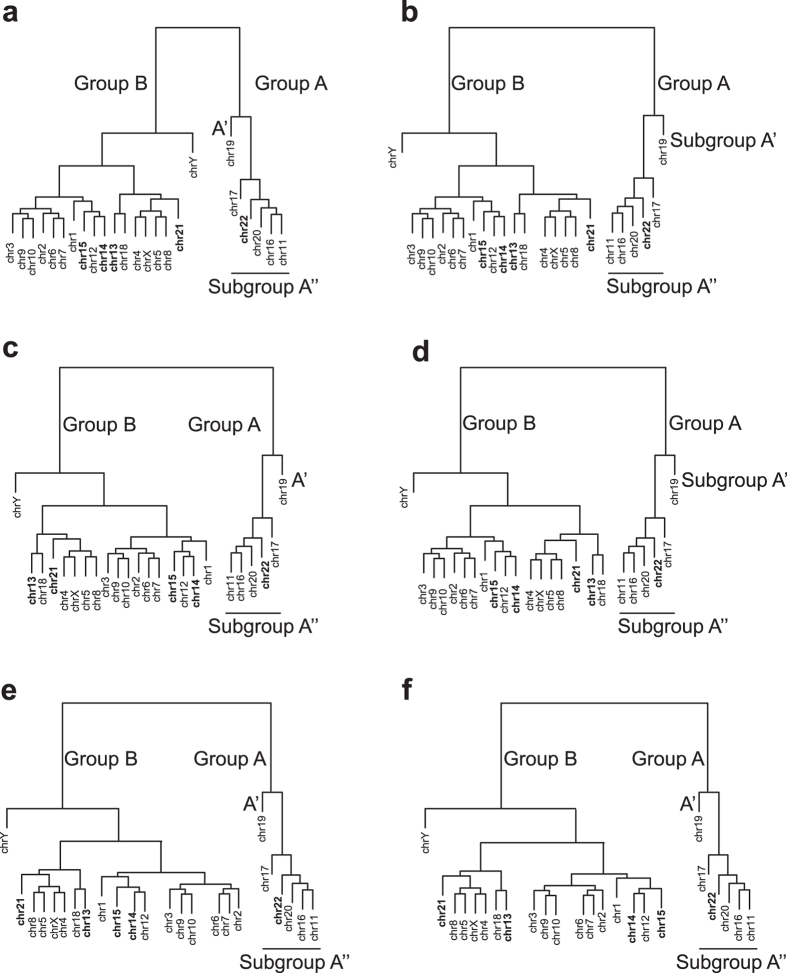
Equivalent hierarchically clustered dendrograms derived from the effective gene density matrix. The suprachromosomal coupling dendrograms derived from the effective gene density matrix for *Homo sapiens* (using coding and noncoding genome) represent unique CT constellations in (**a**–**f**). These six unique CT constellations can be perceived on an abstract 2D plane by consigning chr19 (human chromosome 19) to the nuclear interior, as in (**a**–**f**). The vertical line connotes hierarchy in the effective gene density whose length relates to diversity in suprachromosomal organization as elaborated below. Chr3 placed at the end of multiple branch-points of the dendrogram with respect to chr19 is considered “distant” in the vertical axis as compared to chrY vis-á-vis chr19. The horizontal branch connotes a mother/daughter hierarchical relationship via branch-points (representing CTs) within a given clade, and therefore their absolute lengths have no physical implication where clades may even be revolved around the primary anchorage point defined by chr19 or around other subordinate branch points, thereby giving rise to equivalent dendrograms (**a**–**f**) and representing the plasticity associated with suprachromosomal organization. Similarly, the vertical line connotes hierarchy in the effective gene density whose length relates to diversity in suprachromosomal organization. As in a hypothetical situation, interior and peripheral CTs are denoted as Groups A and B respectively (see Fig. 1). Although chr21 and chrY are Group B CTs and relatively gene poor, the theory supports a constellation with them as interior chromosomes due to neighbourhood CT effects (**a**). A hierarchical and degenerate representation enables the rationalization of chr21 and chrY as interior CTs shown in panel (**a**) (such as in fibroblasts), versus peripheral CTs in (**b**) (such as in lymphocytes).

As normalized PCGC (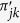) and effective length (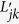) terms are coupled via the effective gene density 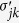 [equation (6)], we investigate its hierarchy in the context of CT arrangements in human interphase nuclei. We collate pairs of CTs with similar average effective gene density to compute a binary tree in an effective gene density space. As effective gene density is a mathematical function of the CT length and gene density, such a binary tree hierarchically clusters CTs based on their extrinsic systems-level attributes. Hence, we used the complete-linkage algorithm (Materials and Method) to compute the hierarchical clustering within the abstract effective gene density space. Our analysis of the human genome led to a segregation of effective gene density from CTs into two primary clusters, Groups A and B (Figs 1b and 3). We represent Group A with chr11, chr16, chr17, chr19, chr20, chr22 and Group B with the remaining 18 CTs (Fig. 3).

### Human chromosome 19 dominates the hierarchy of suprachromosomal effective gene density and dictates spatial CT position in human interphase nuclei

The suprachromosomal effective gene density of human chromosome 19 (chr19) with every other CT represents the entire range of permissible effective gene density values (Fig. 1a). Therefore, chr19 represents the primary hierarchy among all suprachromosomal effective gene density parameters. Interestingly, if we consider a hypothetical pruning of chr19 (from Group A), then the hierarchy of Group A changes completely with respect to Group B. To avoid any algorithmic bias, which may be introduced as an artifact of the clustering method, we re-computed the effective gene density matrix hierarchy using the single-linkage (Fig. S1b), centroid-linkage (Fig. S1c), average-linkage (Fig. S1d), and Mcquity (Fig. S1e) algorithms. We report that chr19 consistently maintains the primary hierarchy as obtained from each of these different linkage methods. Therefore, as chr19 occupies a unique and primary hierarchy among all 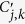, one could represent it at the origin of an abstract effective gene density space, or consign it to the interior of the nuclear volume in the context of a particular CT constellation or even an overall radial distribution of CTs.

Our theoretical analysis essentially tries to capture physical pairing of CTs in an interphase nucleus, whose clustering describes the CT neighbourhood in the context of suprachromosomal organization. As it is not physically possible for any given CT to be nearest neighbour with remaining all (46−1 = 45) CTs at the same time in the same human interphase nucleus, we hypothesize that every CT may putatively be nearest neighbour with any other CT in different interphase nuclei, within a very large ensemble of cells. However, some nearest neighbours may be more frequent than others in a given cell or even in a single cell type specific ensemble, Therefore, following an unbiased approach, all possible chromosome pairs were considered for computing the repository of extrinsic parameters describing them (via PCGC formalism) and the putative “coupling” strengths among them analyzed, via the effective gene density matrix. Once the physical location of the CT with a primary hierarchy is known within the nucleus, our mathematical formulation describes the subsequent hierarchical basis for self-organization of the other CTs within the constellation.

It has already been established that chr19 is at the interior of the human nucleus using fibroblasts^6^,^12^,^13^,^14^,^15^,^16^, lymphocytes^12^,^13^,^15^,^16^ and lymphoblastoids^17^,^18^ cells. Therefore, this localised one-to-one mapping of CTs from an abstract effective gene density space to the real spatial nuclear volume facilitated subsequent spatial arrangement of CTs with respect to each other and with chr19. Hence, a CT constellation may be conceived from the nuclear interior towards the periphery as illustrated in Fig. 1d,e. Our results imply that the primary hierarchy of chr19 in an effective gene density space is essential for the overall non-random and hierarchical spatial arrangement of other CTs in the human nucleus. Therefore, Group A was subdivided into Subgroup A’ (chr19) and Subgroup A” (chr11, chr16, chr17, chr20 and chr22) (Fig. 3).

**Figure 4:**
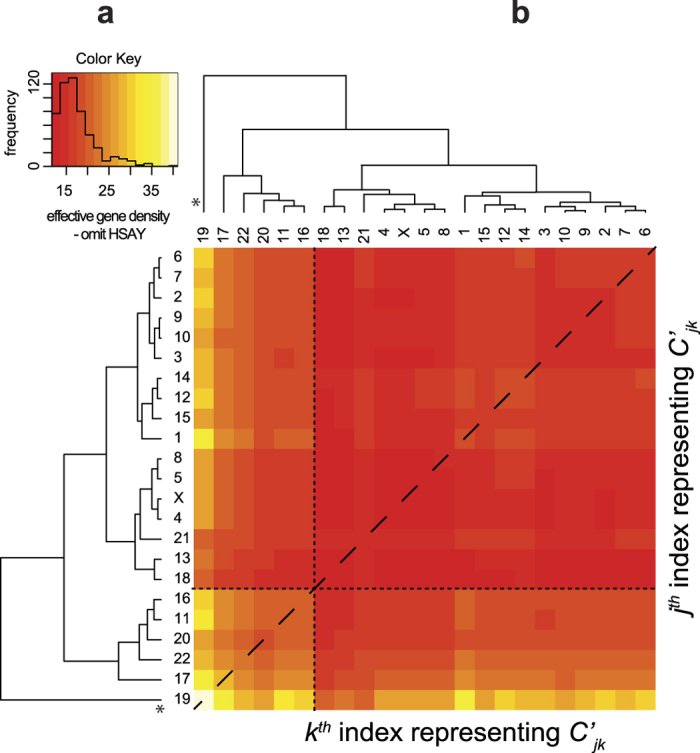
The effective gene density matrix obtained from the (46,XX) diploid female genome. The hierarchy of extrinsic suprachromosomal effective gene density matrix obtained using the intrinsic parameters of all the twenty-three unique chromosomes that represent the diploid 46,XX human female genome. Inset histograms for these panels represent effective gene density colour key. Chromosome 19 with primary hierarchy is marked “*”.

We report that the hierarchy of Subgroup A” was juxtaposed intermediate to chr19 and Group B (Fig. 3), which implies that CTs of Subgroup A” (chr11, chr16, chr17, chr20 and chr22) were constrained to be adjacent to Subgroup A’ (chr19) in a non-random anisotropic constellation. In addition, the hierarchical clustering supports degenerate representations of CTs11 and chr16 in the neighbourhood of other CTs (Fig. 3). The remaining eighteen CTs (Group B), which constituted a dominant fraction, were segregated from Group A’, except for chrY. The dendrogram leaf representing chrY stands out juxtaposed between the six Group A CTs and seventeen Group B CTs (Fig. 3). Noticeably, from Fig. 1b, diversity in effective gene density among 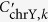 pairs is relatively lower (low variability) compared to corresponding results from 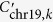 (greater variability). Clearly, in contrast with that of chr19, if chrY were pruned from the dendrogram, the hierarchy of Group A with respect to Group B does not alter (Fig. 1 versus Fig. 4). These results support four important consequences of CT arrangements in human nuclei. Firstly, in a suprachromosomal context, chr19 dominates the mathematical crosstalk, via its relative effective gene density, in the purview of all possible CT constellations. Secondly, it demonstrates that our theoretical basis for suprachromosomal CT constellation is identical in diploid 46,XX female nuclei (Fig. 4) and 46,XY male nuclei (Fig. 1). Thirdly, contingent on near-neighbour effective gene density couplings, the cell type, and the poised state of genes on CTs, we predict that the spatial position of chrY with respect to chr19 can lead to great variability: they can be interior CTs as in fibroblasts (Fig. 3a)^6^ or distantly placed toward the periphery as in lymphocytes (Fig. 3b–f)^12^. Fourthly, and most importantly, the “orphan” nature of chrY, for example, with no homologous partner for crossing over (except with the pseudo-autosomal region in chrX), and its functional specialization for spermatogenesis is also consistent with our systems-level effective gene density hierarchy analyses. The spatially “delocalised” nature of chrY predicted here is indicative of a special evolutionary trajectory of *Homo sapiens* chrY (HSAY), which is very distinct from chrY in *Pan troglodytes* (PTRY)^54^,^55^.

### Corroborating and comparing theoretically obtained results

Next, we corroborate and/or compare all our theoretical results using a five-pronged approach that is based on experimental data, evolutionary comparative genomics and statistical analysis of a hypothetical/thought experiment (*Gedanken* experiment).

#### Comparing the plasticity of CT constellations using FISH results

There is plasticity (ambiguity in preferential positioning) in the CT position of chr1, chr14 and chr21 from FISH studies^6^,^12^,^15^,^16^, even within a clonal population. Typically FISH studies employ a threshold based assignment of fluorescence signal of a CT to a specific spatial zone in the nucleus whose reiterative co-occurrence marks its non-random location within the nucleus and two reports that we have compared here^6^,^12^, have employed a similar threshold-based cut-off: at least 90% signal and up to 10% noise levels to distinguish coloured pixels in images. Another point of caution is in order here because typically CT positioning experiments are carried out using 2D versus 3D FISH approaches. Some of the best-studied examples involving fibroblasts versus lymphocyte nuclear CT analyses have concluded that a high correlation exists between the results of 2D versus 3D FISH analyses. This holds true for both fibroblast and lymphocyte nuclei^6^,^12^. Here, using published reports^6^,^12^, we compiled the inherent variability in preferential (or most likely) CT locations for nine CTs, namely chr1, chr5, chr6, chr8, chr15, chr16, chr20, chr21 and chrY, in lymphocytes and fibroblasts that highlight such an ambiguity (Table 1). A physical basis for this ambiguity in positioning among certain CTs may now be rationalized here using our PCGC model for the first time. We delineated seventeen CTs from Group B (excluding chrY) as largely equidistant and *en masse* from Group A, (primarily in the context of chr19) in effective gene density space. However, due to degenerate configurations, it is plausible that cohorts of CTs in Groups A and B will have an effective gene density-based altered spatial arrangement (for example with respect to chr19) and still maintain effective gene density hierarchy (Fig. 3). Such a hierarchical and degenerate representation has enabled ambiguous yet non-random chromosomal spatial arrangement, which has been experimentally discerned but not rationalized so far. As shown earlier in Fig. 3a, the position of chrY in the dendrogram facilitates its spatial locations in the neighbourhood of six CTs from Group A (largely gene rich CTs) as well as the other seventeen CTs from Group B (largely gene poor CTs) as represented in the equivalent dendrogram representations (Fig. 3). Similarly, it can also be argued that near-neighbour effects will give rise to dissimilar CT neighbourhoods or constellations exemplified by chr21 as shown in Fig. 3a versus 3c, giving rise to contrasting spatial positions (interior versus periphery) in fibroblasts and lymphocytes respectively^6^,^12^ (Fig. 2).

Consider another CT constellation that includes chr11 (Fig. 3b), where we can justify the formalism of neighbourhood CT effects by positioning it with chr1 and chr14 as intermediate to peripheral (Fig. 3c). For example, chr11 in the vicinity of chr1 or chr14 (Fig. 3c) has lower effective gene density, as opposed to chr11 in the vicinity of chr16 or chr20 (Fig. 3a) as CTs couple differently among themselves in these two scenarios (a lower effective gene density neighbourhood occurs if the gene density disparity among them is large and vice versa). Using the same physical basis that justifies various CT constellations, we can represent chr18 or chr2 towards the nuclear interior (Fig. 3d,e) and likewise the sub-cluster of chr12, chr14 and chr15 as shown in Fig. 3f. Clearly spatial positioning of CTs can be very different, particularly when cell-specific instances and poised state of genes are considered. Permutations among CTs with degenerate effective gene density neighbourhoods may have negligible consequence on a pan-nuclear scale. Hence, such degenerate CT permutations may be more feasible as opposed to those involving non-degenerate effective gene density CT neighbourhoods. Therefore, our theory provides the physical basis for diverse anisotropic constellations of CTs at the suprachromosomal level.

#### Corroborating the hierarchy of effective gene density using Hi-C results

Lieberman-Aiden *et al.* have computed inter-CT contact probabilities for normal human cell lines^17^. Specifically they have reported that the small gene-rich CTs such as chr16, chr17, chr19, chr20, chr21 and chr22 preferentially interact with each other. From this group except for chr21, all other CTs match with our results (Table S2). This cluster is represented in our analysis as Group A (Figs 1 and 3). We rationalize that near-neighbour effects in our PCGC theory, as illustrated in Fig. 3a, may lead to the omission of chr21 from the Group A and consigned to Group B in our analysis. In that scenario, chr21 is placed at the edge of Group B CTs, adjacent to Group A. In addition, our Group A also contained chr11, which was not supported by the Hi-C result. Interestingly, in the tethered chromosome capture (TCC) technique, which is a derivative of the Hi-C experiment that sought to improve the signal-to-noise ratio, Kalhor *et al.*^18^ have showed that chr11 is part of an inner cluster of CTs, consistent with our Group A CTs (Table S2). So it is justifiable that the effects of neighbourhood-CTs and cell type-specificity constellations, and the sensitivity of experimental methods dictate how well we resolve the unique from rare constellations. Kalhor *et al.*^18^ computed inter-CTs contact probabilities to generate a dendrogram that was mainly resolved as two large clusters, referred to as Cluster 1 and Cluster 2^18^. The reported Cluster 1 contained 10 CTs, which included chr11, chr16, chr17, chr19, chr20 and chr22, all of which are Group A CTs. However, in addition chr1, chr14, chr15 and chr21 were also part of Cluster 1. In fact, chr1 (the largest chromosome in the human genome) is represented as one that may be localised proximal to Group A CTs in our hierarchy analyses (Fig. 3c), a result consistent with chr1 being part of the inner core CTs^18^. Therefore, we conclude most of our results of constellation plasticity and the hierarchical clustering of CTs have also been supported by these unbiased Hi-C experiments.

#### Corroborating interior CTs using disparate mammalian genomes

**Figure 5:**
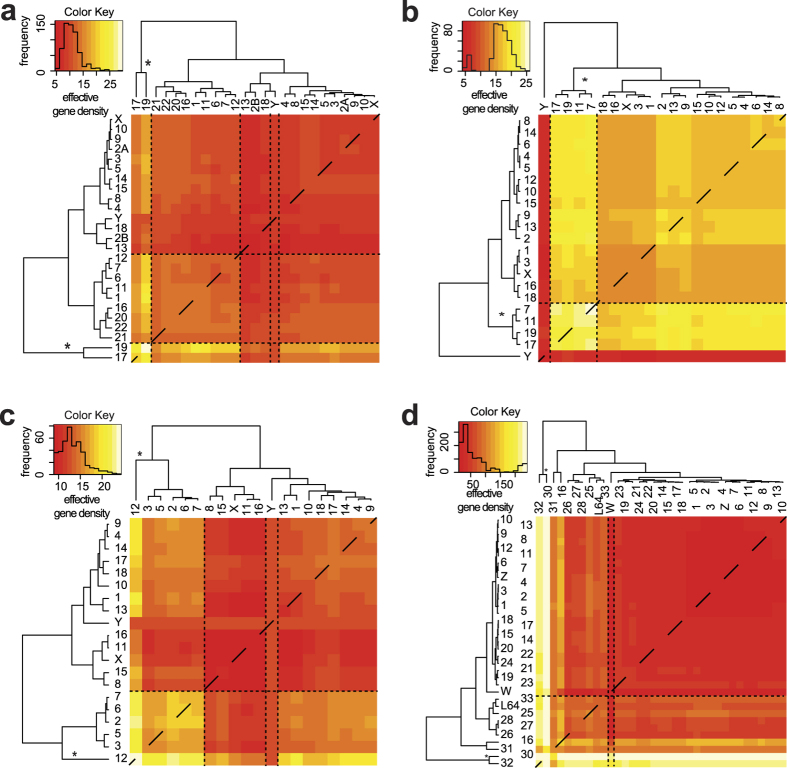
Heatmaps and dendrograms obtained using effective gene density matrix from disparate vertebrate genomes. A heatmap and dendrogram represents the mathematical hierarchy of the effective gene density matrix in diverse genomes that have radial chromosomal arrangement: (**a**) *Pan troglodytes*, (**b**) *Mus musculus*, (**c**) *Sus scrofa* and (**d**) *Gallus gallus*. Panel (d) has been drawn only from available annotated macro- and microchromosomes from NCBI’s Gene database (chr LGE64 is abbreviated as L64). Primary hierarchy interior CT(s) are marked (*). ChrY (depicted as W in panel (d)) is delineated using a pair of dashed vertical lines in each instance. Inset histograms represent colour keys.

Next, we used four different model organisms, which have been shown to exhibit radial-like CT arrangement, to compute CTs with primary hierarchy in the abstract effective gene density space. Using PCGC analysis, we predicted interior CTs in *Pan troglodytes* (Fig. 5a), *Mus musculus* (Fig. 5b), *Sus scrofa* (Fig. 5c) and *Gallus gallus* (Fig. 5d) nuclei. We corroborated our predictions using previously reportedly FISH results for chimpanzee^50^, mouse^51^, pig^52^ and chicken^53^. We apply our theory to these vertebrates as their radial CT arrangement has been previously studied, and predict their inner core CTs. Without using the DNA sequence, we report that PTR17 and PTR19 (in chimpanzee), MMU7 and MMU11 (in mouse), SSC12 (in pig), and GGA30, GGA32, GGA27 and GGA28 (in chicken) occupy the position of primary hierarchy. All of these CTs have been identified as interior CTs^50^,^51^ (Table 1). However, as shown in Table 1, for two species the spatial position of CTs with primary hierarchy have not been mapped using FISH technique. However, we report that they hierarchically cluster to other CTs that have been mapped as interior CTs: pig chromosome SSC12^52^ and chicken microchromosomes GGA30, GGA32^53^,^56^. Next, one can identify the human chromosomes, which have predominant synteny with predicted inner CTs to map the evolution of chromosomes (Table 1). Here, in Table 1 we report that the predicted CTs with primary hierarchy is largely syntenic to HSA19 (human chromosome 19, Group A’ – the CT with overall primary hierarchy), and/or HSA17 (human chromosome 17 – the CT with primary hierarchy in Group A”, and secondary to HSA19). Most interestingly, in each of these studies, the hierarchical clustering of CTs delineates at least two primary clusters. The first cluster has a relatively high and diverse effective gene density (off-white to yellow block diagonal feature) and the second cluster has a much lower but relatively uniform effective gene density (uniformly red block diagonal feature). Such a hierarchical pattern is similar to the high effective gene density block diagonal feature (Group A, Fig. 1b), and the low effective gene density block diagonal feature (Group B, Fig. 1b). As a further corroborative approach, the hierarchical position of human chrY (HSAY) is compared across the vertebrates: a recent ancestral primate: *Pan troglodytes*^57^,^58^ to an evolutionarily distant ancestral vertebrate: *Gallus gallus*^59^. The position of HSAY (Fig. 1b), is juxtaposed between two large CT clusters. However, the position of PTRY (the chrY in *Pan troglodytes*) is embedded in a subordinate CT sub-cluster along with PTR2B (chr2B), PTR13 (chr13) and PTR18 (chr18) (Fig. 5a), implying a significant difference in the suprachromosomal hierarchy. It is interesting that the orphan nature of chrY reported earlier for *Homo sapiens* (Fig. 1a) is also embedded in the effective gene density matrix obtained from *Mus musculus* (Fig. 5b), *Sus scrofa* (Fig. 5c) and *Gallus gallus* (Fig. 5d, with chromosome W) but definitely not in the more recent ancestor *Pan troglodytes* (Fig. 5a). Independent studies based on the genome sequence information have also reported that HSAY significantly differs from PTRY, and also with the computed last common ancestral sequence^55^. Most interestingly, here we have identified this diversity between HSAY and PTRY without using nucleotide sequence information. Thus, the derived physical basis for a suprachromosomal ordering is applicable across diverse eukaryotic species, provided that their overall interphase CT arrangement is radial. We reiterate that our method succeeds in uncovering innate differences in the chrY hierarchy compared to the rest of CTs in a genome as a system-level consequence, without invoking its DNA sequence.

#### Plasticity in the spatial position of nucleolus and nucleolus organizer region in the light of the acrocentric CTs

Nucleolar organizer region (NOR) within the human nucleus is partly constituted by five acrocentric CTs (chr13–15, chr21–22), which are spatially associated with the nucleolus^60^. Based on our effective gene density hierarchical clustering, we just reported that four of these five acrocentric CTs (chr13–15 and chr21) belonged to various subgroups of Group B, whereas chr22 belonged to Group A (Fig. 1a). More importantly, they do not all cluster together in the same clade. The possible plasticity and constraints in the spatial arrangement of these acrocentric CTs (with respect to each other), suggests that the spatial positioning of nucleolus may also exhibit similar plasticity in the context of cell type-specific constraints. Interestingly, the TCC based Hi-C study^18^, which we referred to earlier, has reported that the five acrocentric chromosomes were also differently located in their hierarchy dendrogram (Fig. 6c in ref. 18) because chr14, 15, 21 and 22 were in different subgroups of Cluster 1 but chr13 was in Cluster 2. Moreover, multiple nucleoli associated with a single human nucleus may represent an assortment of NOR between homologous acrocentric CTs. Detailing the assortment of CTs carrying NOR among different nucleoli, if any, is an exciting open biological question. Lastly, we reiterate that our results were compared with the plethora of FISH-based microscopy results of reported CT arrangements in human interphase nuclei^6^,^12^,^13^,^14^,^15^,^16^.

#### Gedanken experiments describe astronomical plasticity in CT positioning

In a *Gedanken* experiment, which is a hypothetical thought experiment, we considered the nuclear volume to be divided into five shells of nearly equal 2D sub-nuclear annular regions, a formalism which was motivated from unbiased high-throughput imaging studies for the human nuclei^6^,^12^,^15^,^16^,^61^. If we permuted nearly equal number of CTs in each of the five hypothetical concentric shells, 10 in one and 9 in each of the others, and estimated the total number of possible permutations, without considering any biological or stearic restrictions, then the total number of unique CT constellations was a tall order 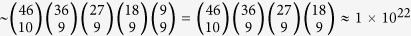. As all CTs do not occupy identical volumes and they may spread across one or more such hypothetical shells, clearly this Gedanken exercise does not accurately represent the CT distribution observed across shells but only depicts that an astronomical number of CT constellations may exist, hinting at inherent organizational plasticity. Even if CTs spread across one or more shells, the conclusion that astronomical number of CT constellations exists does hold. Next, consider the reported marginal probability distribution (as opposed to the joint-probability distribution) of various CTs. We estimate the fraction of cell population, where each of the twenty-four distinct CTs is hypothetically localised at its most probable concentric shell. For simplicity, if we consider the marginal probability of individual CT occurrence as 40% (a value that is similar in magnitude to gene-rich CTs), then a marginal probability for all CTs in their most preferred shell is a very small percentage = 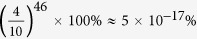. It is plausible that the experimentally determined joint probability (not marginal probability) for some suprachromosomal units would be significantly higher compared to their marginal probability. However, our hypothetical approximations suggest that there may be no unique constellation that can represent 1% of the population from the same cell type. Instead, we can rationalize that such highly likely constellations can well be more than 5–10 times the second most populous constellation. Therefore, there may exist subgroups of similar CT constellations that predominate (Fig. 3), and yet represent a very high degree of plasticity within any given CT constellation, which is an important issue that has remained largely unaddressed by the scientific community.

Now, we estimate the percentage of population of forty-five CTs occupying their most likely shell in various nuclei and one of them (the forty-sixth one) occupying a least likely shell (say with hypothetical probability of 5%). The percentage of such nuclei will be ~6 × 10^−18^% of the entire population, which is lower in magnitude lower that the previous computation. Thus, a nontrivial fraction of nuclei may support CT constellations wherein one CT is in an unlikely location but all others occupy preferred positions (Fig. 3a versus Fig. 3b). Therefore, an empirical basis, which actually predates the full sequencing and annotation of the noncoding human genome, that suggests (i) “gene-rich” CTs, or (ii) small-sized CTs occupy the nuclear interior, falls short in rationalizing a physical basis for a constellation of forty-six CTs. To further strengthen our hypothesis, we contrast the trend in the marginal probability of finding two leading gene-rich CTs, chr19 and chr17, in the interior versus the periphery of the nucleus. We do this by computing the marginal probability of two and four of the gene rich CTs and comparing the results obtained from the two CTs that are localised to the nuclear periphery, chr5 and chr18 (Fig. S2). Clearly, the marginal probabilities may report a significantly lower probability of simultaneous positioning of all gene rich CTs, or all gene poor CTs in the interior or the periphery of the nucleus.

### Human chromosome 19 dominates the effective gene density hierarchy of the protein-coding/noncoding genome

**Figure 6:**
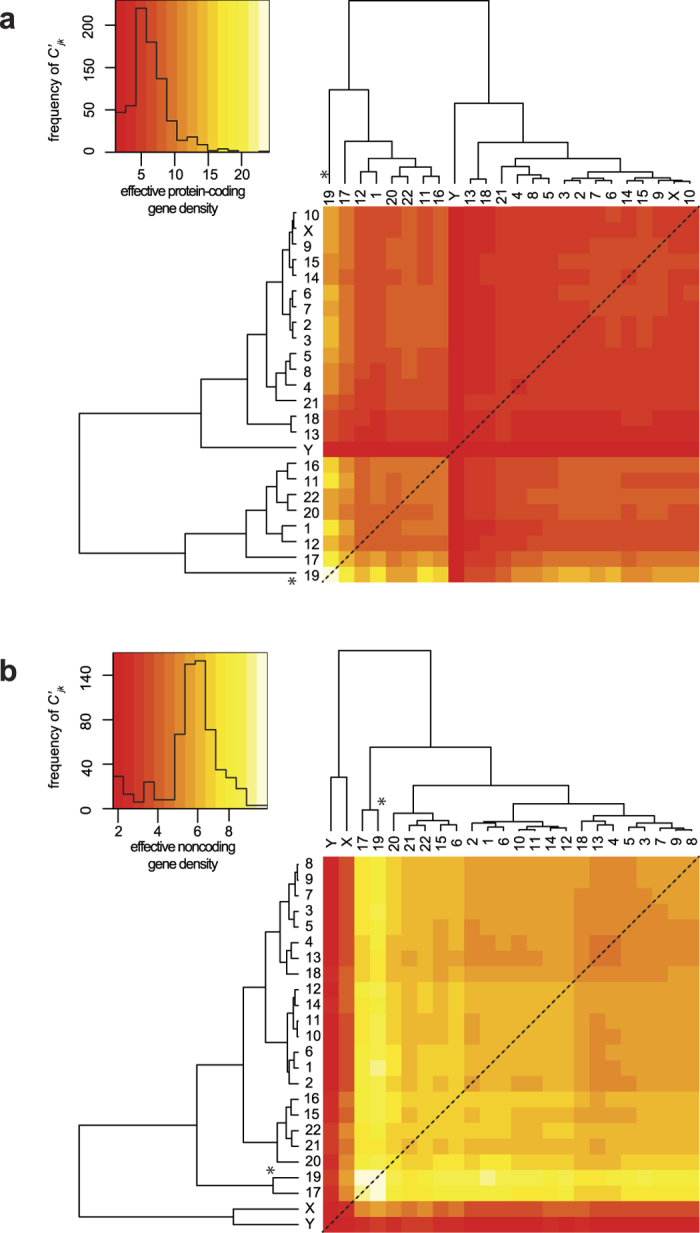
The effective gene density matrix derived from the coding/noncoding human genome. The hierarchy of the extrinsic suprachromosomal effective gene density matrix is derived from the exclusive protein-coding genome (**a**), and the exclusive noncoding genome (**b**). Inset histograms represent colour keys. Primary hierarchy interior CT(s) are marked (*).

We wanted to contrast the exclusive suprachromosomal hierarchy from the protein-coding and noncoding genome. First, we re-derived the effective (protein-coding) gene density matrix using the intrinsic parameters from the human protein-coding genome. Hence, the effective number of genes and effective gene density expressed in equations (1), (2) and (4) at best represented 2% of the human genome. The heatmap representative of the effective gene density for the coding genome is shown in Fig. 6a. Although, the hierarchical clustering differs from the one given in Fig. 1, there are notable common features: (i) chr19 has primary hierarchy, followed by chr17 exactly as in Fig. 3, (ii) chrY is juxtaposed between two large subgroups of CTs (identical to Groups A and B in Fig. 3). The differences between the dendrograms from Figs 1 and 6a are: (i) all tertiary sub-clusters differ, (ii) chr1 and chr12 are clustered with chr19, chr17, chr20 and chr22 (Fig. 6a).

Following this, we computed an effective gene density matrix using the intrinsic parameters of the human noncoding genome. Its hierarchy (Fig. 6b) in effective gene density is different from that of the coding genome (Fig. 6a), or the full genome (Fig. 1). The features differentiating dendrograms are: (i) the two most noncoding gene dense chromosomes: chr17 and chr19 cluster together, and the two sex chromosomes branch off from them, (ii) chrX and chrY cluster together, segregated from the autosomes (Fig. 6b) and are most subordinate in the hierarchy – least effective noncoding gene density with all other CTs and lastly, (iii) entire autosomal effective gene density is largely high (off-white to yellow to light orange in the heatmap), demarcated from relatively lower values (dark-orange to red in the heatmap) of sex chromosomes, as a bimodal distribution in the histogram (Fig. 6b). Our overall results suggest that within an effective gene density space, a differential mix of coding and noncoding parts of the genome might significantly alter effective gene density based suprachromosomal hierarchy. Interestingly, results with the coding genome effective gene density map is almost at par with the whole genome analysis, where we have extensively showed that chr19 maintained its status of primary hierarchy (Figs 1, 3 and S1).

### Saliency of hierarchy as obtained from the effective gene density

In a given biological context, differential levels of activated and inactivated genes exist per chromosome, which may clearly influence both the intrinsic properties of a CT as well neighbouring CTs via extrinsic couplings. During chromosomal translocations, the physical values of intrinsic parameters of affected CTs are also altered. An *in silico* analysis was implemented to estimate the incremental change in intrinsic parameters, which altered the original hierarchy and degeneracy in the effective gene density. To assess the saliency and sensitivity in changing intrinsic parameters of this complex system, we tuned one parameter at a time keeping others constant, generating hypothetical surrogate genomes. We computed the minimum decrement in the number of genes for each chromosome that led to an altered dendrogram in Fig. 3. The change could be a reorganization of the hierarchy within at least one subgroup. The results of such sensitivity analysis for all chromosomes are presented in Table S3. We observed that the decrement of genes required for a change in dendrogram for each chromosome was variable. In summary, a sensitivity analysis substantiates that primarily small changes (~1%) and few large changes (~10%) in the number of genes within a chromosome adversely affect the original hierarchy and degeneracy of CTs in an effective gene density space. However, in any case, the decrement computed as a percent change in the genome was rather small (0.1–1.0%). This demonstrates that hierarchy dendrogram is a sensitive construct representative of the whole human genome organization, with regard to a CT constellation in the nucleus. Our saliency analysis also supports the inherent plasticity among constellations. Clearly, omission of a hundred genes is not feasible in a biological context, but this *in silico* experiment helps uncover the level of sensitivity of an effective gene density hierarchy in a pan-nuclear setting.

## Discussion

At an empirical level, barring exceptions, intrinsic parameters (coding gene density or/and chromosome size) have been implicated to predict CT arrangement from FISH studies^12^,^13^,^14^,^15^,^16^. It has also been reported that relatively small-sized CTs, independent of their protein-coding gene density, are localised to the nuclear interior, but larger ones are at the periphery^14^,^15^,^16^, again barring exceptions. As a specific example, inconsistency in the most probable CT location between two widely studied cell types, fibroblasts and lymphocytes were considered (Table S1)^6^,^12^. Here, we instantiated how intrinsic parameters do not completely represent the physical basis of a spatial CT arrangement in a densely packed milieu of human nuclei. (1) Spatial positions of chr21 and chrY, both small-size CTs with low gene density, are inconsistent in fibroblast and lymphocyte nuclei. Both CTs occur largely at the nuclear interior in fibroblasts but at the periphery in lymphocytes. (2) Inconsistency in the spatial locations of seven other CTs (chr1, chr5, chr6, chr8, chr15, chr16 and chr20) in fibroblasts versus lymphocytes. (3) Consistent (or weakly consistent) consensus locations obtained for chr10, chr11 and chr14 in these two cell types. Interestingly, unlike most other CTs, these do not have a preferred spatial territory in the nuclei (interior versus intermediate versus periphery). (4) Most importantly, in the realm of protein-coding gene density or size-based segregation, the threshold of high versus low intrinsic parameters that segregate interior versus peripheral CTs is qualitative or at best empirical, and lacks mathematical rigor because individual CT constellations have not yet been mapped yet. On the other hand, our theory supports an overall unique non-random hierarchical arrangement of CTs (a CT constellation), which arises along with permissible degeneracy and positional anisotropy, a result that has been experimentally confirmed using Hi-C techniques^17^,^18^,^19^. Moreover, our theoretical formalism derived at the pan-nuclear scale, provides the mathematical basis for a self-organized CT arrangement due to nearest-neighbour CT effects within the nucleus, substantiating a previously proposed hypothesis^62^,^63^.

Matrix algebra has been extensively used to describe systems-level information that may be innocuous at times, such as in molecular phylogenetics, involving sequence evolution as illustrated via phylogenetic trees. In an unrelated study, a systems-level approach has computed differential gene expressions obtained from microarray data by deriving “eigengenes” and “eigenarrays” via vectors and matrices to identify coexpressed over-active and under-active regulatory genes in genome-wide studies^64^. While in the cited study, matrix formalism has been used to decompose the expression data, a converse approach has been used in our current study since we unify the intrinsic chromosomal parameters with one another to uncover the systems-level crosstalk among CTs.

We have demonstrated that the extrinsic suprachromosomal couplings (effective gene density) derived using our systems-level theory substantiates the hierarchy, degeneracy, and constrains spatial CT arrangement. We surmise that such biological hierarchy ensures a self-organization in human nuclei with forty-six CTs. In fact the hierarchical plasticity of effective gene density, by virtue of different suprachromosomal neighbourhoods, emphasizes that such systems are dynamic and require coupling and decoupling among themselves to sustain a radial arrangement that can self-organize. Moreover, there is compelling evidence from Hi-C studies to suggest cell-to-cell variations in topological conformation of CTs^19^, and that CTs may also have preferential orientation with respect to landmarks such as the nuclear envelop^65^ supporting neighbourhood influence (mathematically demonstrated here in our paired chromosome’s gene count formalism). In the current study we have investigated three paradigms that involved (i) the whole genome, protein-coding and noncoding parts of the genome (Figs 1 and 6), (ii) protein-coding part of the genome exclusively (Fig. 6a), and (iii) noncoding part of the genome exclusively, only as a mathematical exercise (Fig. 6b). All three paradigms support the hierarchy of extrinsic effective gene density matrix, which led us to unravel the physical principle of a self-organized assembly of CTs. We have approximated the suprachromosomal mathematical crosstalk by using the first-order interactions or nearest-neighbour CTs (Materials and Methods). Interestingly, chrY has the most subordinate hierarchy in each of these three paradigms, reinforcing that the physical basis for CT constellations at the suprachromosomal level is identical for the 46,XY and the 46,XX diploid genomes. Although we identified patterns of similarity and dissimilarity in the extrinsic suprachromosomal couplings for these three paradigms, we obtained results that support an overall hierarchy and plasticity of suprachromosomal constellations. The uniqueness in the results of the three paradigms discussed above suggests instances when the three paradigms may coexist in varying degrees as dictated by the biology of the nucleus, whose detailed analysis is beyond the scope of our current study. We reiterate that the abstract effective gene density space defined by the differential mix of the coding versus noncoding human genome might significantly alter the hierarchy of suprachromosomal coupling in a cell type-specific manner. If one was to extend this model to incorporate three nearest-neighbour CT interactions, additional genomic constraints will be described, implying much tighter constraints among genomic parameters. Moreover, this theory (in its current form) does not distinguish between maternal and paternal homologous chromosomes as it assumes and invokes an identical number of genes and chromosomal lengths for each of those homologs, but is flexible enough such that it can be modified to incorporate genomic variants (chromosomal translocations or also large deletions in either maternal or paternal chromosomes) to study the altered suprachromosomal organization.

The primary hierarchy of chromosome 19 is noteworthy here because it encodes for more than double the genome-wide average number of genes (coding as well as noncoding). The large clustered gene families, which correspond to high G + C content, CpG islands and density of repetitive DNA indicate a chromosome that is rich in functional importance^66^ and evolutionary history. Independent comparative sequence analyses reveal that there exist large conserved syntenic blocks from mammals^67^,^68^ to even fish^69^ and birds^59^. The preeminent dominance of chr19 in the overall hierarchy is evident in the whole genome as well as in the protein-coding genome effective gene density analyses. And most interestingly, this dominance spreads across other species where chromosomes syntenic to HSA19 occupy that analogous position. It is to be noted that our current method uncovered all these features without invoking any chromosomal sequence information.

In conclusion, the effective gene density matrix of the human genome unifies intrinsic parameters of CTs and mathematically constraints their spatial arrangement. The anisotropy associated with effective gene density, in our *in vivo* representation, provides a physical basis for a non-random and self-organized radial CT arrangement in a densely packed nuclear milieu. We have corroborated and rationalized our findings with available experimental data, and substantiated our hypothesis in disparate mammalian species that extrinsic effective gene density matrix constrains spatial CT arrangement in interphase nuclear milieu. Based on the observed corroboration, we conclude that mathematical hierarchy among CT’s deciphered in the current study closely matches the physical spatial hierarchy among CTs, which compels us to equate coupling of CTs to physical clustering of the same that eventually forges a non-random organization at suprachromosomal level. We also hypothesize that a high plasticity of CT constellations, within a given clonal population of cells, might set up cellular heterogeneity, as reported in single cell Hi-C studies^19^. However, the cell type-specific nuclear architectural components, via lamin and other nuclear-matrix mediated interactions^9^,^10^,^11^, contribute towards the maintenance of CT arrangement across cell divisions. Finally, we also applied this systems-level theory across disparate mammalian species with annotated genomes, and identified the interior core CTs in their nuclei. In the general evolutionary context, our results provide a geometry-based perspective to the CT arrangement (*in vivo* evolution of CT constellations in 3D) within the vertebrate nuclei.

## Methods

### Intrinsic parameters of the human genome

The total number of annotated genes per chromosome, and their respective lengths were obtained from National Center of Biotechnology Information (NCBI) Gene database and Mapviewer portal (release 107) for the human genome. These parameters are available in Table S4.

### A systems-level representation of the human genome using matrix algebra

For notational brevity, 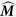 denotes a matrix, |***C***〉 denotes a column vector, and 〈***R***| denotes a row vector, such that 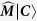, 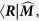 and 〈***R***|***C***〉 denote an inner product and |***C***〉〈***R***| denote an outer product. All representative labels ***C*** and ***R*** are parameters (either intrinsic or extrinsic parameters) that qualitatively describe these vectors. This is a conventional notation in physics, which has also been used in biological sciences^64^.

For a systems-level theory, we represent the number of genes (coding and noncoding) and the length of chromosomes (traditional intrinsic parameters), for the *N* (=24) distinct chromosomes of the human genome, as two *N* dimensional column vectors denoted by 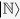 and |**Λ**〉 respectively. The coupling of these two vectors in the abstract vector space is via a *N* × *N* matrix. Since we used identical macroscopic intrinsic parameters for pairs of homologous chromosomes, our formalism does not support a physical basis special position or localisation of paternal versus maternal chromosomes.

### Unification of macroscopic intrinsic parameters in the human genome

We denoted the twenty-four different chromosomes of the human genome (chr1, chr2,… chr21, chr22, chrX and chrY representing chromosome 1, 2 and so on) as *C*_*j*_ (where 1 ≤ *j* ≤ *N* = 24). Mathematically, the average gene density of *j*^*th*^ chromosome (*C*_*j*_) is represented as a “coupling” term *d*_*j*_ in a scalar equation *n*_*j*_ = *d*_*j*_*L*_*j*_, where *n*_*j*_ is the total number of genes (including protein-coding and noncoding genes) and *L*_*j*_ is the length of *C*_*j*_. Traditionally, *n*_*j*_ is considered a parameter that only involves the *j*^*th*^ chromosome, independent of any other *C*_*k*_, which may not necessarily be a nearest neighbour of *C*_*j*_. Therefore, equations involving all intrinsic chromosomal couplings may be collectively represented as 
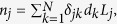
 were *δ*_*jk*_ is the Kronecker delta function (=1 for indices *j* = *k*, and = 0 for *j* ≠ *k*). This Kroneer delta formalism enabled the inclusion of a null value to gene density coupling terms for all dferent chromosomes except *C*_*j*_. As we showed in Results, this formalism enabled us to represent the macroscopic intrinsic parameters of *C*_*j*_ as being influenced by another *C*_*k*_, and delineate genome-level constraints, which were otherwise not apparent. Using this mathematics, we formulated a theory where all intrinsic parameters of different chromosomes constitutively coupled together and then derived their systems-level crosstalk. It did not matter whether or not the CTs were physically nearest-neighbours, because this formalism is set up such that the set of macroscopic chromosomal parameters is a self-consistent solution of a set of simultaneous linear equations. Therefore, to facilitate this systems-level approach in an *in vitro* setting, we proposed 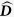 as a diagonal *N* × *N* matrix with intrinsic gene density (*d*_*j*_) as its diagonal elements (real eigenvalues of 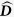). It must be noted that intrinsic parameters are experimentally obtained from cytogenetic as well as sequencing efforts under laboratory conditions and they represent an *in vitro* milieu. Therefore, these parameters may not accurately represent typical systems biology of the *in vivo* milieu that nuclei manifest. For brevity, we denote the set of basis vectors for the *in vitro* or diagonal representation as 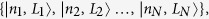 labeled by the intrinsic parameters: total gene count per chromosome (*n*), its length (*L*) in megabase (Mb) and a subscript *j* for referencing *C*_*j*_ (Intrinsic chromosomal parameters are provided in Table S4). Now, a systems-level formalism in a *N*-dimensional vector space using a *N* ×*N in vitro* coupling matrix is presented as a matrix equation:


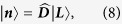


where,


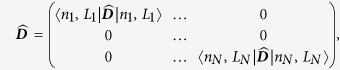


such that the intrinsic gene density 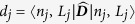 (megabase pair Mbp^−1^ units). Here, |***n***〉 and |***L***〉 are *N* × 1 column vectors whose components represent the *in vitro* intrinsic parameters, namely the number of coding genes and chromosome length. As the *in vitro* gene density space does not describe inter-chromosomal couplings, the hierarchical nature of extrinsic gene density remains implicit.

### Extrinsic parameters of the human genome

Now, we mathematically describe extrinsic chromosomal parameters in an *in vivo* context. We used the intrinsic parameters from all the unique (*N* = 24) human chromosomes (22 autosomes and X, Y chromosomes) to quantify the mathematical crosstalk among the 46 CTs (diploid genome) within the human nucleus. Now, systems-level coupling among them may be represented in a *N* dimensional *in vivo* vector space defined by a *N* × *N* Hermitian matrix 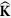. This space is defined by a set of *N* basis vectors 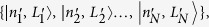 labeled by extrinsic systems-level parameters: effective gene count (*n*^′^) and effective length (*L*^′^) in Mbp, along with subscripts that denote labels from column and row vectors. Next, analogous to the *in vitro* model (equation (8)), we represent an abstract effective gene count and effective length as vectors denoted by 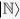 and |**Λ**〉 respectively. We posit:


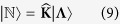



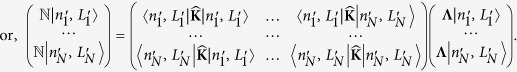


We hypothesize that the systems-level coupling of CTs is commutative and associative, such that it may be represented by matrix 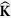, which is a real, symmetric Hermitian matrix. We hypothesize that this coupling matrix is due to all possible nearest-neighbour suprachromosomal coupling of *C*_*j*_ with *C*_*k*_. Hence in equation (9) the original generic coupling matrix 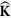 may be approximated to the first-order by 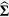, where


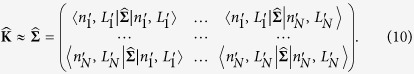


The elements of 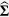 are the extrinsic coupling coefficients, and physically they represent the suprachromosomal mathematical crosstalk. Next, we derive these extrinsic couplings using intrinsic chromosomal parameters: number of genes (*n*_*j*_), chromosomal length (*L*_*j*_), gene density (*d*_*j*_), along with *N* scalar equations *n*_*j*_ = ∑_*k*_*δ*_*jk*_*d*_*k*_*L*_*j*_, with *d*_*j*_ > 0, *L*_*j*_ > 0, where *δ*_*jk*_ is the Kronecker delta function. To explicitly derive the obscure suprachromosomal “mixing” (which we refer to as biological crosstalk), we represent 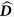 in the *in vivo* vector space. This deconstruction is obtained, using the Spectral Theorem, as a symmetric *N* × *N* matrix 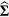 (from equation (10)) such that:


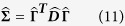



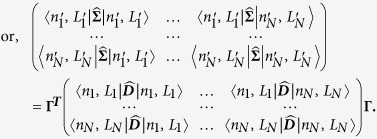


Here, the *j* row and *k* column element of 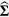 is denoted as 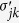, in megabase pair (Mbp) units, (where 
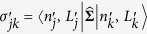
), and 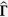 is a *N* × *N* orthogonal matrix (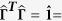 unitary matrix). The *bra* and *ket* vectors represents labels that pertain to macroscopic chromosomal parameters and must not be confused as dummy indices intended for summation.

### Hierarchical clustering of effective gene density

To deconstruct the hierarchy of the effective gene density matrix, we used five different hierarchical clustering algorithms implemented in the R software-package (hclust function that is based on Fortran code contributed to STATLIB by Murtagh). The different algorithms implemented were (i) complete linkage, (ii) single linkage, (iii) centroid linkage, (iv) average linkage and (v) Mcquity.

## Additional Information

**How to cite this article**: Fatakia, S. N. *et al.* Systems-level chromosomal parameters represent a suprachromosomal basis for the non-random chromosomal arrangement in human interphase nuclei. *Sci. Rep.* **6**, 36819; doi: 10.1038/srep36819 (2016).

**Publisher’s note**: Springer Nature remains neutral with regard to jurisdictional claims in published maps and institutional affiliations.

## Supplementary Material

Supplementary Information

## Figures and Tables

**Table 1:** Interior CTs predicted using hierarchical clustering of effective gene density matrix.

Species (Abbreviation)	Reference corroborating CT position	Predicted interior CT(s)	Dominant synteny with HSA chromosome
*Pan troglodytes* (PTR)	ref. 50	PTR17, PTR19	HSA17^§^, HSA19^§^ ref. 50
*Mus musculus* (MMU)	ref. 51	MMU7, MMU11	HSA19^§^, HSA17^§^ Mouse Genome Sequencing Consortium^68^
*Sus scrofa* (SSC)	ref. 52^$^	SSC12^$^	HSA17^§^ ref. 70
*Gallus gallus* (GGA)	ref. 53^¶^ ref. 56	GGA30^¶^, GGA32^¶^, GGA27^¶^, GGA28^¶^	NA^¶¶^ International Chicken Genome Consortium^59^ HSA17^§^, HSA19^§^

CT(s) with primary hierarchy in effective gene density are located in the nuclear interior and influence CT neighbourhoods forming a non-random CT constellation. HSA, PTR, MMU, SSC and GGA are standard identifiers used to represent the human (*Homo sapiens*), chimpanzee (*Pan troglodytes*), mouse (*Mus musculus*), pig (*Sus scrofa*), and chicken (*Gallus gallus*) chromosomes respectively. The interior CTs: HSA19 and HSA17 have the highest and second highest hierarchy in effective gene density matrix (Fig. 1) and synteny relations with diverse species are with respect to these CTs^§^. The spatial position of SSC12 has not been reported though it has been reported that porcine chromosomes: SSC2, SSC3, SSC4, SSC5, SSC6 and SSC7 are interior CTs^52^, which we have reported to hierarchically cluster with SSC12^$^ (Fig. 5c). Habermann *et al.*^53^ have shown that microchromosomes (such as GGA30, GGA32, GGA27, GGA28) are at the nuclear interior and macrochromosomes are at the periphery in *Gallus gallus* fibroblast cells^¶^. The predicted CT with primary hierarchy (GGA30) currently has ten annotated coding and noncoding genes and synteny information is not available. However, as microchromosomes GGA27 and GGA28 are in same hierarchical cluster as GGA30 and GGA32 and because their synteny information was previously known^59^, therefore we have reported using the same^¶¶^.
