# Supplementary material for: Systems-level chromosomal parameters represent a suprachromosomal basis for the non-random chromosomal arrangement in human interphase nuclei
Source: Sci Rep. 2016 Nov 15;6:36819. doi: 10.1038/srep36819 (PMC5109186; doi:10.1038/srep36819)
Supplement: Supplementary Information [file srep36819-s1.pdf]

Supplementary Information

Systems-level chromosomal parameters represent a suprachromosomal basis for the non-random chromosomal arrangement in human interphase nuclei

Sarosh N. Fatakia[1]*, Ishita S. Mehta[1,2] and Basuthkar J. Rao[1]*

[1]Department of Biological Sciences, Tata Institute of Fundamental Research, Mumbai, Maharashtra 400005, India.

[2]UM-DAE Centre for Excellence in Basic Sciences, Biological Sciences, Kalina campus, Santacruz (E), Mumbai, Maharashtra 400098, India.

* Corresponding authors: Dr. Sarosh N. Fatakia, Email: sarosh.fatakia@tifr.res.in and Professor Basuthkar J. Rao, Email: bjrao@tifr.res.in

Phone number: +91-22-22782606, Fax number: +91-22-22804610

Figure S1

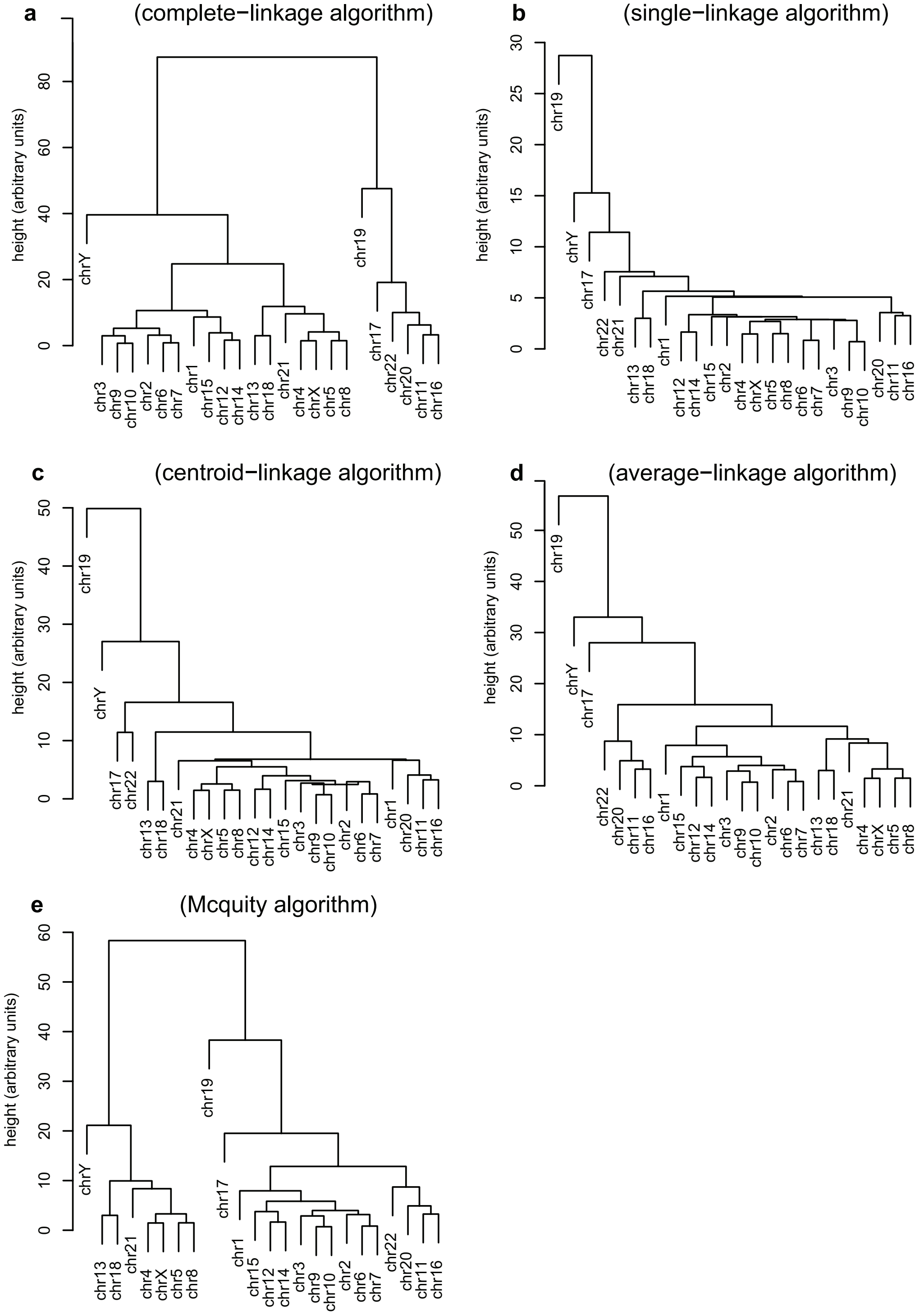

Figure S1. Hierarchical clustering of extrinsic effective gene density matrix computed using different algorithms. Dendrograms reveal the primary hierarchy of HSA19 (chr19), as obtained for CT pairs using the systems-level paired chromosome's gene count model, using five algorithms: complete-linkage (**a**), single-linkage (**b**), centroid-linkage (**c**), average-linkage (**d**), and Mcquity method (**e**).

Figure S2

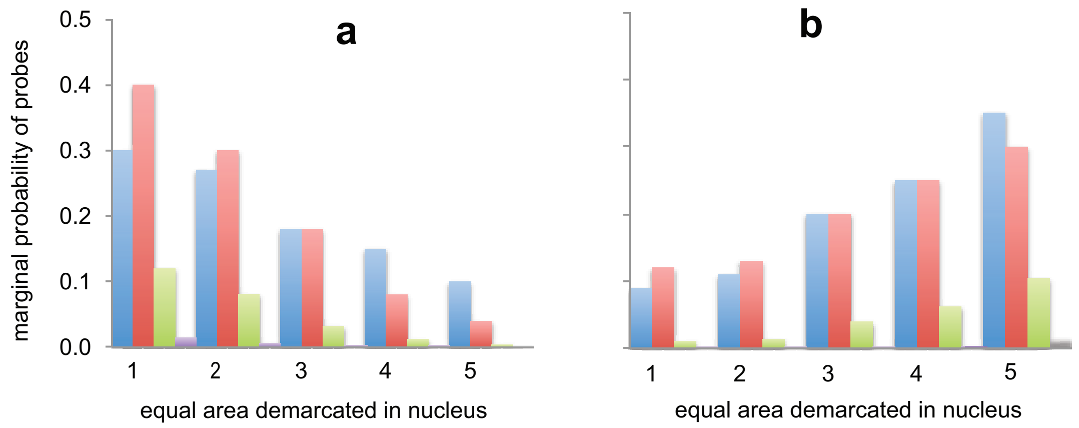

Figure S2. Bar graphs representing the spatial position of selected CTs in human interphase fibroblast nuclei. Marginal probability for aggregates of CTs in the nuclear spatial volume, which has been projected on to equal area annular 2D surface, is revealed as shells 1 – 5; shell 1 being toward the nuclear periphery and shell 5 at the nuclear interior. (**a**) Describes the probability distribution of probes from solo CTs chr18 (blue) and chr5 (red), the marginal probability of jointly finding one chr18 and one chr5 (green) (two chromosomes) and the marginal probability of jointly finding both homologs of chr18 and chr5 (violet) (four chromosomes). (**b**) Describes the probability distribution of probes from two "gene-rich" CTs, chr19 (blue) and chr17 (red), the marginal probability of jointly finding one chr19 and one chr17 (green) and the marginal probability of jointly finding both homologs of chr19 and chr17 (violet). The probability of finding individual probes was obtained from Mehta *et al.,* 2013.

Table S1. Preferential location of CTs obtained from normal human fibroblast and lymphocyte nuclei. A summary of dominant CT positions obtained from a population of human dermal fibroblast and lymphocyte nuclei using independent experimental microscopy studies. Experimental results from microscopy data were obtained for lymphocytes from Figure 3 as in [¶]Boyle, *et al.* 2001, and for fibroblasts from Additional file 1 as in [§]Mehta, *et al.* 2013**.** This table is a summary of those results. A 2D projection of FISH image from a nucleus was compartmentalized into five equal area annular shells, and maximal signal from the probed CT was ascribed to one of those annular shells: 1- 5 (Shells 1-2 were designated as peripheral, shell 3 as intermediate, and shells 4-5 as interior, see Fig. S2). FISH images from a clonal population of cells yields a distribution for CT position across these shells (Fig. S2). The most probable position for that CT will correspond to the shell where its distribution peaks. Here we compare peak distributions from lymphocytes and fibroblasts, for chr1-22, X and Y, from Boyle, *et al.* 2001 and Mehta, *et al.* 2013.

**Chr ID**	**Lymphocyte nuclei[¶]**	**Fibroblast nuclei[§]**	**Summary**
**chr1**	Interior – Intermediate	Intermediate – periphery	***Inconsistent***
**chr2**	Periphery	Periphery	Consistent
**chr3**	Periphery	Periphery	Consistent
**chr4**	Periphery	Periphery	Consistent
**chr5**	Intermediate	Periphery	***Inconsistent***
**chr6**	Interior –Intermediate – Periphery	Periphery	***Inconsistent***
**chr7**	Periphery	Periphery	Consistent
**chr8**	Periphery	Interior – Intermediate – Periphery	***Inconsistent***
**chr9**	Periphery	Periphery	Consistent
**chr10**	Intermediate	Interior – Intermediate – Periphery	*Weakly consistent*
**chr11**	Intermediate – Periphery	Periphery	*Weakly consistent*
**chr12**	Periphery	Periphery	Consistent
**chr13**	Periphery	Periphery	Consistent
**chr14**	Interior – Intermediate – Periphery	Interior – Intermediate – Periphery	Consistent
**chr15**	Intermediate	Periphery	***Inconsistent***
**chr16**	Interior – Intermediate	Intermediate – Periphery	***Inconsistent***
**chr17**	Interior	Interior	Consistent
**chr18**	Periphery	Periphery	Consistent
**chr19**	Interior	Interior	Consistent
**chr20**	Periphery	Interior - intermediate	***Inconsistent***
**chr21**	Interior – intermediate – Periphery	Interior	***Inconsistent***
**chr22**	Interior	Interior	Consistent
**chrX**	Periphery	Periphery	Consistent
**chrY**	Periphery	Interior	***Inconsistent***

Table S2. Corroboration of theoretical results with those obtained from independent Hi-C experiments. Corroboration of predicted inner core human CTs (Group A in our PCGC analysis) using results from two independent high-throughput studies. Column 2 represents the Group ID of individual CTs as obtained from our paired chromosome gene count study (Fig. 1a and Fig. 3). In column 3, the validity of our results of CT clustering is reported with respect to results in Figure 2B from Lieberman-Aiden *et al.* 2009. In column 4, our PCGC results of CT clustering (Group A) was validated with inner core Cluster 1 CTs in Figure 6C from Kalhor *et al.* 2012. Similarly, the Group B CTs were validated with respect to Cluster 2 from the same reference.

chrID	Hierarchy Group ID	Hi-C corroboration	TCC corroboration
**chr1**	B	Valid	Invalid
**chr2**	B	Valid	Valid
**chr3**	B	Valid	Valid
**chr4**	B	Valid	Valid
**chr5**	B	Valid	Valid
**chr6**	B	Valid	Valid
**chr7**	B	Valid	Valid
**chr8**	B	Valid	Valid
**chr9**	B	Insignificant	Valid
**chr10**	B	Valid	Valid
**chr11**	A	Invalid	Valid
**chr12**	B	Valid	Valid
**chr13**	B	Valid	Valid
**chr14**	B	Insignificant	Invalid
**chr15**	B	Insignificant	Invalid
**chr16**	A	Valid	Valid
**chr17**	A	Valid	Valid
**chr18**	B	Valid	Valid
**chr19**	A	Valid	Valid
**chr20**	A	Valid	Valid
**chr21**	B	Invalid	Invalid
**chr22**	A	Valid	Valid
**chrX**	B	Valid	Valid
**chrY**	B	Not available	Not available

Table S3. Results from sensitivity studies using intrinsic chromosomal parameters. The limiting values of gene reduction per chromosome that leads to an altered dendrogram computed using effective gene density.

chr ID	Minimum gene reduction for dendrogram change		Aprox % altered genome
	Num. genes (per chr)	*% genes* (per chr)	
**chr1**	300-400	8%-9%	1.0%
**chr2**	< 40	< 1%	0.1%
**chr3**	30-60	1%-2%	0.1%
**chr4**	70-100	4%-5%	0.2%
**chr5**	70-80	2%-3%	0.2%
**chr6**	200-250	7%-8%	0.5%
**chr7**	100-110	4%-5%	0.2%
**chr8**	70-100	3%-4%	0.2%
**chr9**	100-120	5%-6%	0.2%
**chr10**	70-80	3%-4%	0.2%
**chr11**	180-230	3%-4%	0.5%
**chr12**	80-90	2%-3%	0.2%
**chr13**	140-150	11%-12%	0.3%
**chr14**	40-50	1%-2%	0.1%
**chr15**	30-50	2%-3%	0.1%
**chr16**	10-20	<1%	0.1%
**chr17**	50-80	2%-3%	0.1%
**chr18**	40-50	5%-6%	0.1%
**chr19**	70-100	3%-4%	0.2%
**chr20**	20-30	2%-3%	0.1%
**chr21**	40-50	5%-6%	0.1%
**chr22**	40-50	4%-5%	0.1%
**chrX**	100-110	5%-6%	0.2%
**chrY**	50-60	10%-11%	0.1%

Table S4. Intrinsic parameters of chromosomes in the human genome. Intrinsic chromosomal parameters were obtained from NCBI's Gene and Mapviewer database.

chr ID	Number of genes per chr (n_j)	Chromosome length (L_j) (in Mbp)
**chr1**	4763	250
**chr2**	3598	242
**chr3**	2780	198
**chr4**	2251	190
**chr5**	2392	182
**chr6**	2825	171
**chr7**	2596	159
**chr8**	2003	145
**chr9**	2132	138
**chr10**	2040	134
**chr11**	2804	135
**chr12**	2396	133
**chr13**	1326	114
**chr14**	1949	107
**chr15**	1719	102
**chr16**	1842	90
**chr17**	2326	83
**chr18**	938	80
**chr19**	2420	59
**chr20**	1243	64
**chr21**	725	47
**chr22**	1129	51
**chrX**	1972	156
**chrY**	496	57
